# Identification of Signature Genes Associated With Invasiveness and the Construction of a Prognostic Model That Predicts the Overall Survival of Bladder Cancer

**DOI:** 10.3389/fgene.2021.694777

**Published:** 2021-09-13

**Authors:** Yang He, Yongxin Wu, Zhe Liu, Boping Li, Ning Jiang, Peng Xu, Abai Xu

**Affiliations:** ^1^Department of Urology, Zhujiang Hospital, Southern Medical University, Guangzhou, China; ^2^Department of Urology, The First People’s Hospital of Kashgar Prefecture, Kashgar, China

**Keywords:** bladder cancer (BLCA), invasiveness, weighted gene co-expression network analysis (WGCNA), nomogram, tumor immune microenvironment

## Abstract

**Background:** Bladder cancer has become the tenth most diagnosed cancer worldwide. The prognosis has been shown to differ between non-muscle invasive bladder cancer (NMIBC) and muscle invasive bladder cancer (MIBC). We aimed to identify signature genes that are associated with the invasiveness and survival of bladder cancer and to identify potential treatments.

**Methods:** We downloaded gene expression profiles of bladder cancer from the Gene Expression Omnibus database to identify differentially expressed genes and perform weighted gene co-expression network analysis. Functional enrichment was analyzed by GO and KEGG analyses. Hub genes were identified from the significant module. Another dataset was also acquired to verify the expression of hub genes. Univariate and multivariate Cox regression analyses were applied to the dataset downloaded from The Cancer Genome Atlas database. Risk scores were calculated and the effect was evaluated by Kaplan-Meier survival analysis. A nomogram was constructed and validated using training and testing samples, respectively. Analysis of the tumor immune microenvironment was conducted with the CIBERSORT algorithm.

**Results:** In total, 1,245 differentially expressed genes (DEGs) were identified. A distinct module was identified that was significantly correlated to invasiveness. The genes within this module were found to be significantly associated with extracellular exosomes, GTPase activity, metabolic pathways, etc. Three hub genes (VSIG2, PPFIBP2, and DENND2D) were identified as biomarkers of invasiveness; two of these (PPFIBP2 and DENND2D) were closely associated with prognosis. The risk score was regarded as an independent prognostic factor. The nomogram was associated with acceptable accuracy for predicting 1- and 5-year overall survival. The infiltrating levels of resting NK cells, activated natural killer (NK) cells, CD8^+^ T cells, activated memory CD4^+^ T cells, and T follicular helper cells, were significantly higher in the group with lower risk scores. The group with higher risk scores showed predominant infiltration by regulatory T cells (Tregs).

**Conclusion:** We successfully identified three signature genes related to invasiveness and constructed a nomogram of bladder cancer with acceptable performance. Differences suggested by risk scores between groups of patients showing diverse patterns of immune cell infiltration may be beneficial for selecting therapeutic approaches and predicting prognosis.

## Introduction

Bladder cancer (BLCA) has become the tenth most commonly diagnosed type of cancer worldwide, with approximately 573,000 new cases per year, a morbidity of 3.0%, and 213,000 deaths; the mortality rate associated with BLCA is 2.1% (Sung et al., 2021). In clinical diagnosis, approximately 75% of patients with bladder cancer are diagnosed with non-muscle invasive bladder cancer (NMIBC); the others are diagnosed with muscle invasive bladder cancer (MIBC); diagnosis is made according to whether the tumor invades the muscular layer of the bladder (Avgeris et al., 2018). The postoperative recurrence rate of NMIBC exceeds 70%; approximately 15% of these patients progress to MIBC. The postoperative recurrence rate of patients with MIBC can exceed 50% after radical cystectomy (RC), and many patients can die from this disease (Kotolloshi et al., 2021; Zhang et al., 2021). Because of the different prognoses and biological pathways underlying NMIBC and MIBC, a range of different treatment strategies may be required (Nouhaud et al., 2021). Therefore, it is crucial and beneficial to identify genes or features that are of prognostic value and to establish a prognostic model that could identify potential treatments and predict prognoses.

**FIGURE 1:**
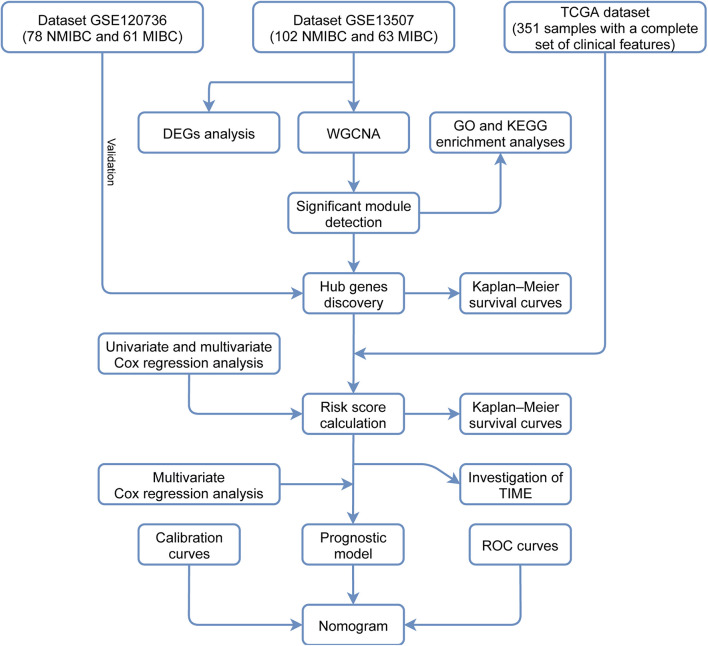
A flowchart depicting the entire study process.

In this study, we analyzed mRNA expression data relating to BLCA from the Gene Expression Omnibus (GEO) database using differential gene expression analysis and weighted gene co-expression network analysis (WGCNA). Then, we identified the genes in the significant module that was most relevant to invasiveness and analyzed these genes using pathway and functional enrichment analyses. We also identified survival-associated hub genes and clinical signatures to predict the prognoses of patients with BLCA and developed a robust prognostic model to help direct treatment strategies and decision-making in the clinical treatment of BLCA patients. Finally, we investigated the diversity of tumor immune cell infiltration between different groups of patients with different risk scores. Figure 1 shows a flowchart of the entire study process.

## Materials and Methods

### Data Download and Processing

The GSE13507 microarray dataset features the gene expression profile of patients with BLCA (Lee et al., 2010) and contains 102 NMIBC samples and 63 MIBC samples. This dataset was downloaded from the GEO database.^1^ We also downloaded the GSE120736 dataset (Song et al., 2019) which contains 78

NMIBC samples and 61 MIBC samples; this was used to verify the expression profiles of the signature genes. We also downloaded a standardized RNA-seq fragments per kilobase per million mapped reads (FPKM) dataset for BLCA from The Cancer Genome Atlas (TCGA) database^2^ in order to construct a reliable prognostic model and investigate the tumor immune microenvironment (TIME).

### The Identification of DEGs Between NMIBC and MIBC Samples

We utilized the ‘‘limma’’ package downloaded from Bioconductor^3^ in R to analyze differentially expressed genes (DEGs) between NMIBC and MIBC samples. In order to select significant DEGs, we first normalized gene expression levels and then set the adjusted *P*-value to < 0.05 and the | log2 fold change| to > 0.5 as thresholds.

### Weighted Gene Co-expression Network Analysis

We used all of the genes in the GSE13507 dataset for WGCNA; this was performed using the “WGCNA” R package (Langfelder and Horvath, 2008). Our aim was to explore the relationships between significant expression modules and invasiveness. We created a sample clustering tree to detect and eliminate an outlier and set the soft thresholding power value to 9 in order to obtain a scale-free network. The resulting adjacency matrix derived from the gene expression set was then converted to a topological overlap matrix (TOM) for module clustering. We set 30 as the minimum number of genes in each module, and similar modules were merged with a threshold cut-off of 0.25. Next, we generated a hierarchical clustering dendrogram; distinct colors were assigned to diverse branches to reveal different modules. The vital clinical trait was then integrated into the eigengene network as an auxiliary node to explore the connection between the trait and the modules. Module-trait associations were then evaluated by analyzing the correlation between module membership (MM) and gene significance (GS). Modules that were highly correlated with invasiveness were selected and extracted to perform subsequent analysis.

### Enrichment Annotation Analysis

Genes identified in the crucial module were then analyzed by Gene Ontology (GO) enrichment analysis and Kyoto Encyclopedia of Genes and Genomes (KEGG) pathway analysis. For this, we used the Database for Annotation, Visualization, and Integrated Discovery (DAVID)^4^ an adjusted *P* < 0.05 was considered significant.

### Hub Genes

To ensure quantity and accuracy, genes in the most significant module (with | MM| > 0.86 and | GS| > 0.42), as determined by WGCNA, were recognized as hub genes. Kaplan-Meier survival analyses were also adopted to determine the differences in overall survival (OS) between groups of hub genes when expressed at high and low levels.

### Identification of Prognostic Signature Genes

We used a TCGA dataset with a complete set of clinical features (*n* = 351) to identify genes that were relevant to prognosis. Univariate Cox regression analysis was applied to explore the prognostic value of hub genes. After filtration, genes with *P* < 0.1 were selected for multivariate Cox regression analysis to evaluate the interactions between prognosis-related genes; this was carried out with the “survival” package in the R environment.

### Establishment of a Prognostic Model

Next, a prognostic risk score model was developed based on prognosis-associated genes, expression levels, and coefficients. The risk score was calculated using an established formula (Sullivan et al., 2004), as follows:

$$\mathrm{Risk}\,\mathrm{Score}=\sum_{i=1}^{n}{\text{Coefficient}}_{i}\times {\text{Expression}}_{i}$$

Based on the median risk score, we separated all samples from TCGA dataset into two different groups. We carried out multivariate Cox regression analysis again and calculated hazard ratios (HRs) to identify the independence of the risk score for predicting overall survival.

### Construction and Validation of a Nomogram

Samples from TCGA dataset (with a complete set of clinical features) were divided into training (*n* = 252) and testing (*n* = 99) cohorts randomly. A nomogram derived from the training dataset was constructed by the “rms” package in R with the following clinical features: age, gender, risk score, T stage, and N stage. We created calibration plots to examine the predictive performance of the nomogram. A receiver operating characteristic (ROC) curve was derived from the testing cohort and used to check the accuracy of the nomogram based on a prognostic model; this was performed with the “survivalROC” package in R.

### Investigation of the Tumor Immune Microenvironment

TIME analysis was applied to samples from TCGA dataset; this was carried out with the “CIBERSORT” analytic tool. We determined the proportions of 22 different tumor-infiltrating immune cells; *P* < 0.05 was considered to be the level of statistical significance (Newman et al., 2015). We also used the Wilcoxon rank-sum test to detect significant differences in the proportions of immune cell infiltration between low- and high-risk groups of patients.

### Statistical Analysis

R software (version 4.0.3), and a range of tools within the R environment, were used for statistical analysis. We employed univariate and multivariate Cox regression analyses to determine prognostic factors. Kaplan-Meier curves were utilized to compare the OS of different groups, and statistical significances were verified with the log-rank test. Two groups of independent non-parametric samples were evaluated by the Wilcoxon rank-sum test.

## Results

### A Comparison of DEGs Between NMIBC and MIBC Samples

**FIGURE 2:**
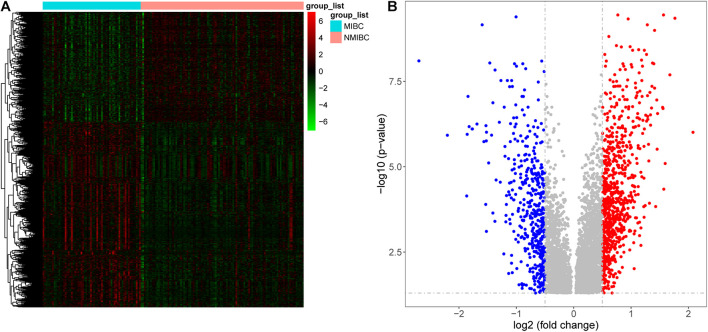
Differentially expressed genes between patients with non-muscle invasive bladder cancer (NMIBC) and muscle invasive bladder cancer (MIBC) displayed as a heatmap **(A)** and a volcano map **(B)**.

Samples from the GSE13507 dataset were normalized and separated according to invasiveness. We identified 1245 DEGs (780 upregulated genes and 465 downregulated genes) using specific cut-off criteria (adjusted *P* < 0.05 and | log2 fold change| > 0.5) (Figures 2A,B).

### The Construction of Weighted Gene Co-expression Network

**FIGURE 3:**
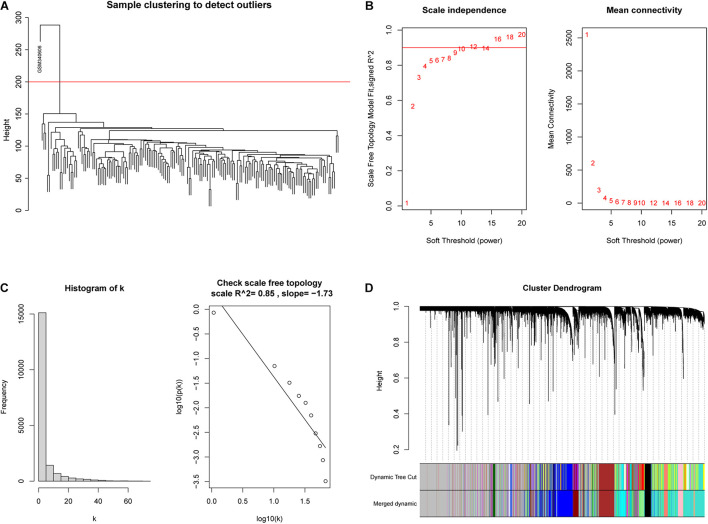
Construction of a weighted co-expression network. **(A)** An outlier (GSM340606) was detected and eliminated by sample clustering. **(B)** β = 9 was selected as the soft threshold. **(C)** Validation of the scale-free topology network. **(D)** The dynamic tree cutting and merging method resulted in the identification of 24 modules.

**FIGURE 4:**
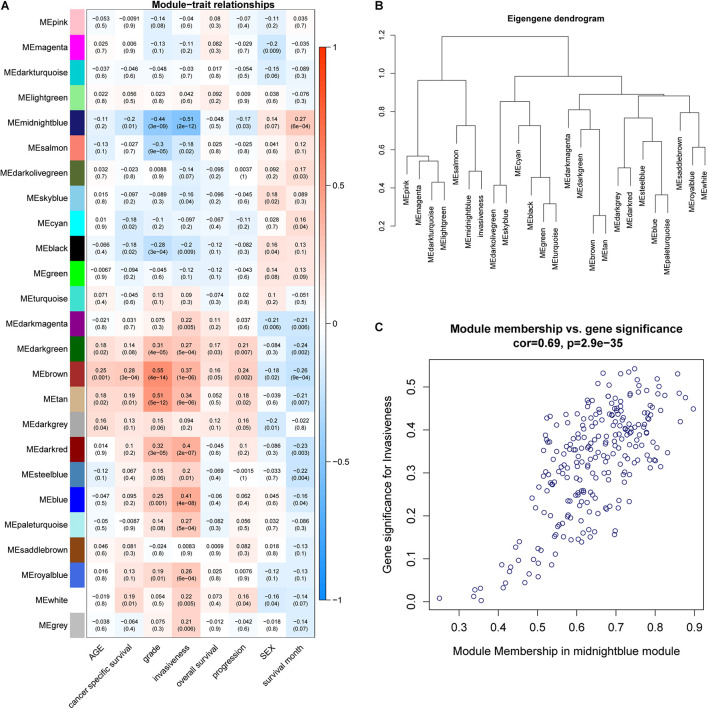
**(A)** A heatmap was created to display the relationships between modules and traits; correlation coefficients and *P*-values are also shown for each module. **(B)** Invasiveness was integrated into the eigengene network to explore the connection between the trait and modules. **(C)** Correlation between module membership (MM) and gene significance (GS).

WGCNA was adopted on samples from the GSE13507 dataset in order to identify genes related to invasiveness. A sample clustering tree was obtained, and an outlier (GSM340606) was detected and eliminated (Figure 3A). Next, we needed to set an appropriate soft threshold; power values ranged from 1 to 20 and a power (β) of 9 was selected to obtain a scale-free network (Figure 3B). The scale-free topology fitting index *R*^2^ reached 0.85 (Figure 3C), thus fulfilling the requirements of scale-free topology. TOM was transformed from an adjacency matrix for clustering modules. Using the dynamic tree cutting and merging method and taking 0.25 as the cut-off point and 30 as the minimum number of genes, we merged similar modules. This strategy ultimately revealed 24 modules from 18,575 genes (Figure 3D). Correlation factors were calculated and then displayed as a heatmap (Figure 4A). The midnight-blue module, containing 240 genes (Supplementary Table 1), were highly correlated with invasiveness (correlation coefficient =–0.51; *P* = 2 × 10^–12^) and grade (correlation coefficient = −0.44; P = 3 × 10^–9^). The trait was then rescaled, using MIBC as the reference; the connection between the invasiveness and each module is shown in Figure 4B and Supplementary Figure 1. There was a strong correlation between the midnight-blue module and phenotype (correlation coefficient = 0.69; *P* = 2.9 × 10^–35^), as shown by Figure 4C.

### Function and Pathway Enrichment Annotation Analysis of the Significant Module

**FIGURE 5:**
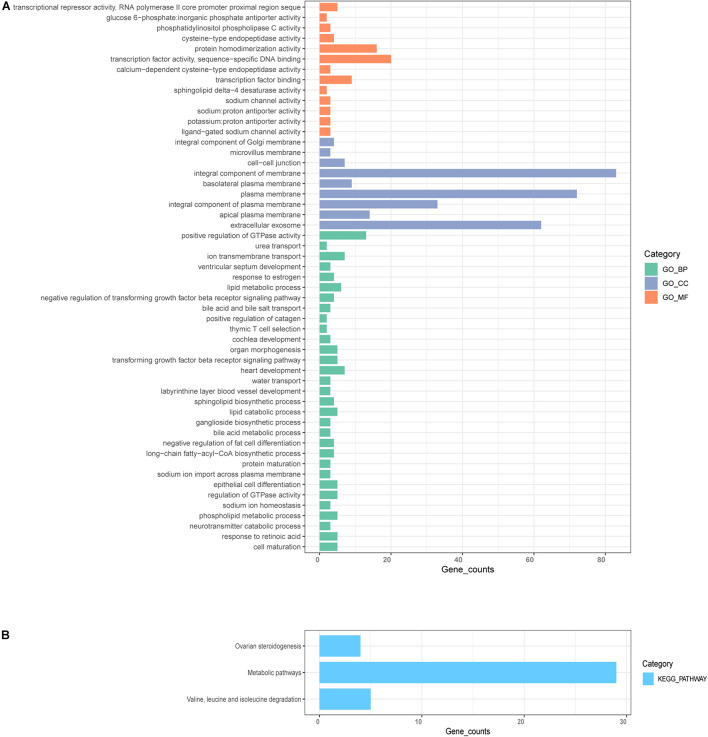
GO and KEGG enrichment analysis. **(A)** GO analysis for genes in significant modules, including biological process (BP), cellular component (CC), and molecular function (MF). **(B)** KEGG enrichment analysis for genes in the significant modules.

Next, we employed GO and KEGG analyses to explore the function and pathway enrichment of the genes involved. The genes in the midnight-blue module were significantly related to protein homodimerization activity, integral component of Golgi membrane, extracellular exosome, lipid metabolic process, epithelial cell differentiation, positive regulation of GTPase activity, negative regulation of transforming growth factor beta receptor signaling pathway, and thymic T cell selection, etc. (Figure 5A, *P* < 0.05). KEGG pathway analysis identified significant enrichment in metabolic pathways; valine, leucine and isoleucine degradation; and ovarian steroidogenesis (Figure 5B, *P* < 0.05).

### The Identification of Hub Genes From the Significant Module

**FIGURE 6:**
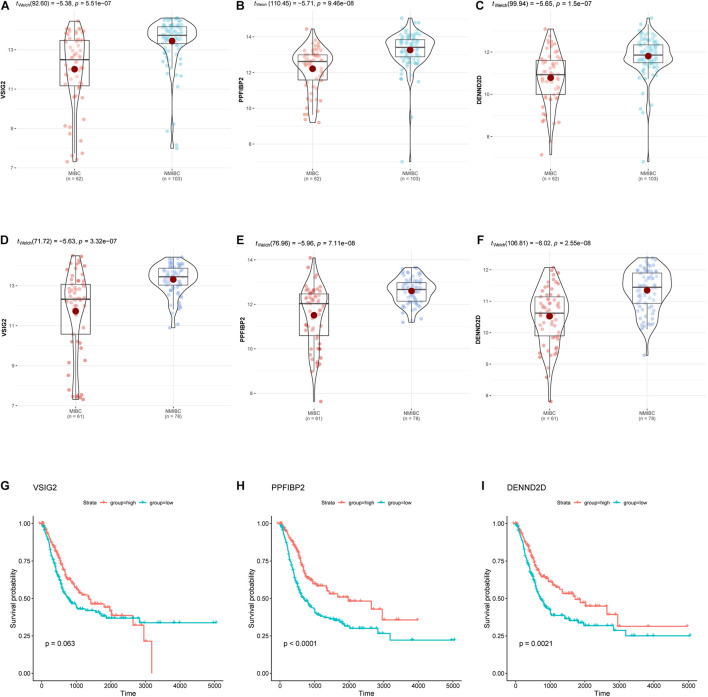
The expression of hub genes (*VSIG2*, *PPFIBP2*, and *DENND2D*) in the GSE13507 dataset **(A–C)** and the GSE120736 dataset **(D–F)**. Kaplan-Meier survival curves for patients with different expression levels of hub genes **(G–I)**. The expression levels of groups were classified according to the median gene expression value.

Hub genes were selected using specific criteria (| MM| > 0.86, and | GS| > 0.42). Three genes were identified: *VSIG2*, *PPFIBP2*, and *DENND2D* (Supplementary Table 2). The expression levels of hub genes in the NMIBC and MIBC groups were visualized as violin plots (Figures 6A–C) using the “ggstatsplot” R package and validated with the GSE120736 dataset (Figures 6D–F); this analysis demonstrated good levels of consistency. The clinicopathological characteristics of patients in the two GEO cohorts are shown in Table 1. Kaplan-Meier survival curves were used to demonstrate the differences in OS between the low- and high- expression groups of hub genes (Figures 6G–I).

**TABLE 1:** Basic clinical characteristics of patients in the GEO datasets.

Variables	GSE13507	GSE120736	*P*-value
Patients	165	139	
**Type**			
NMIBC	102 (61.8)	78 (56.1)	0.313
MIBC	63 (38.2)	61 (43.9)	
**Gender**			
Female	30 (18.2)	24 (17.3)	0.835
Male	135 (81.8)	115 (82.7)	
**Size**			
<3 cm	69 (41.8)	58 (41.7)	0.776
≥3 cm	89 (53.9)	80 (57.6)	
NA	7 (4.2)	1 (0.7)	
**Tumor number**			
Single	98 (59.4)	88 (63.3)	0.198
2–7	45 (27.3)	48 (34.5)	
>8	21 (12.7)	3 (2.2)	
NA	1 (0.6)		
**Stage**			
Ta	24 (14.5)	37 (26.6)	0.676
T1	78 (47.3)	41 (29.5)	
T2–T4	63 (38.2)	61 (43.9)	

### The Identification of Prognostic Signature Genes

**FIGURE 7:**
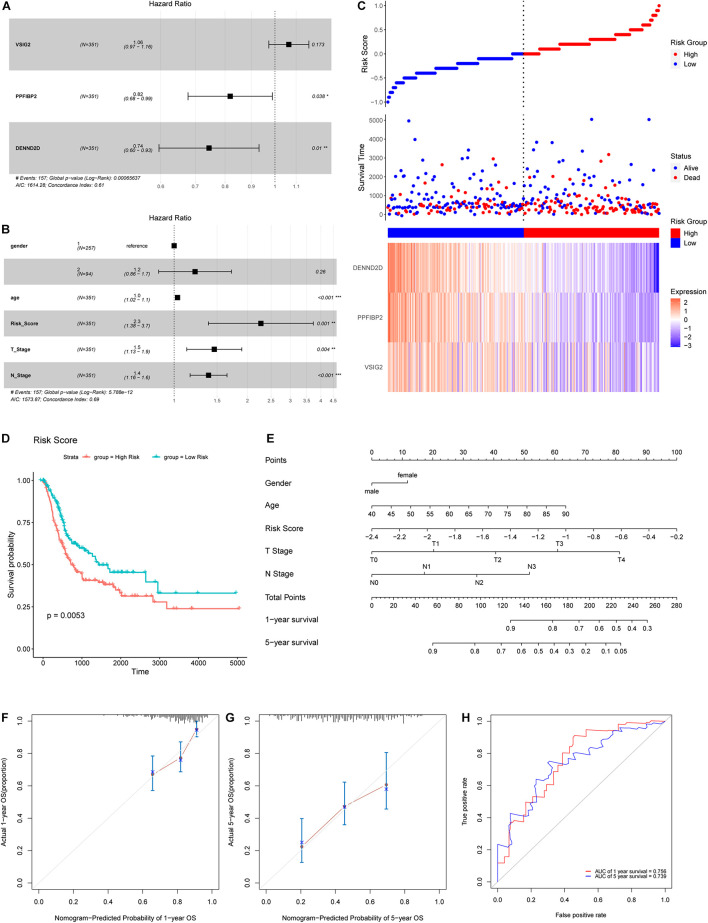
**(A)** A Forest map of hub genes, as determined by multivariate Cox regression analysis. **(B)** A Forest map of risk score and other clinical features, as determined by multivariate Cox regression analysis. **(C)** Risk score distribution, survival status, and an expression heatmap for the three hub genes. **(D)** Kaplan-Meier survival curves for patients in the low- and high-risk groups. **(E)** Nomogram for predicting the 1- and 5-year OS of patients with BLCA. **(F,G)** A calibration curve of the constructed nomogram for predicting 1- and 5-year OS. **(H)** The time-dependent ROC curves for the constructed nomogram using the test data.

Hub genes extracted from the midnight-blue module were then subjected to univariate Cox regression (Table 2). The exclusion criterion was set to *P* > 0.1. Multivariate Cox regression was then applied to the hub genes that passed the exclusion criterion (Figure 7A). The clinical characteristics of patients in TCGA cohort used for analysis are shown in Supplementary Table 3. *PPFIBP2* and *DENND2D* were identified as the hub genes that had the most influence on prognosis.

**TABLE 2:** Univariate Cox regression analysis of hub genes.

Hub genes	Beta	HR (95% CI for HR)	Wald	*P*-value
VSIG2	–0.055	0.95 (0.89–1)	3.5	0.062
PPFIBP2	–0.21	0.81 (0.71–0.92)	10	0.0016
DENND2D	–0.33	0.72 (0.6–0.86)	13	0.00 035

### Establishment of a Prognostic Model Based on Hub Genes

Next, we calculated the risk score for each sample used expression levels of hub genes and coefficients, as follows: Risk Score = (0.06219 × *VSIG2*) + (−0.20005 × *PPFIBP2*) + (−0.29484 × *DENND2D*). The risk score, based on invasiveness, was regarded as an independent prognostic factor for survival, as demonstrated (Figure 7B). According to the median risk score in TCGA cohort, we separated patients into different groups (Figure 7C). The overall survival of low-risk patients was significantly longer than that of high-risk patients, as indicated by survival analysis (Figure 7D).

### Construction and Validation of a Nomogram for Predicting Prognosis of Patients With Bladder Cancer

To predict the prognosis of patients with BLCA, we developed a nomogram using the training cohort from TCGA dataset (Figure 7E). Consequently, 1- and 5-year OS can be estimated according to the total number of points; the risk score had the greatest weighing in this calculation. A patient with a lower risk score, T stage, N stage, or age, has a higher likelihood of a better prognosis. 0.67375977 was found as the C-index for the nomogram. Acceptable consistency between nomogram predictions and actual 1- and 5-year OS was determined by calibration curves (Figures 7F,G). In addition, we also developed time-dependent ROC curves for the established nomogram using the testing cohort (Figure 7H); the area under the curve (AUC) values for 1- and 5-year OS were 0.756 and 0.739, respectively.

### Association of Risk Score With Immune Cell Infiltration in the Tumor Microenvironment

**FIGURE 8:**
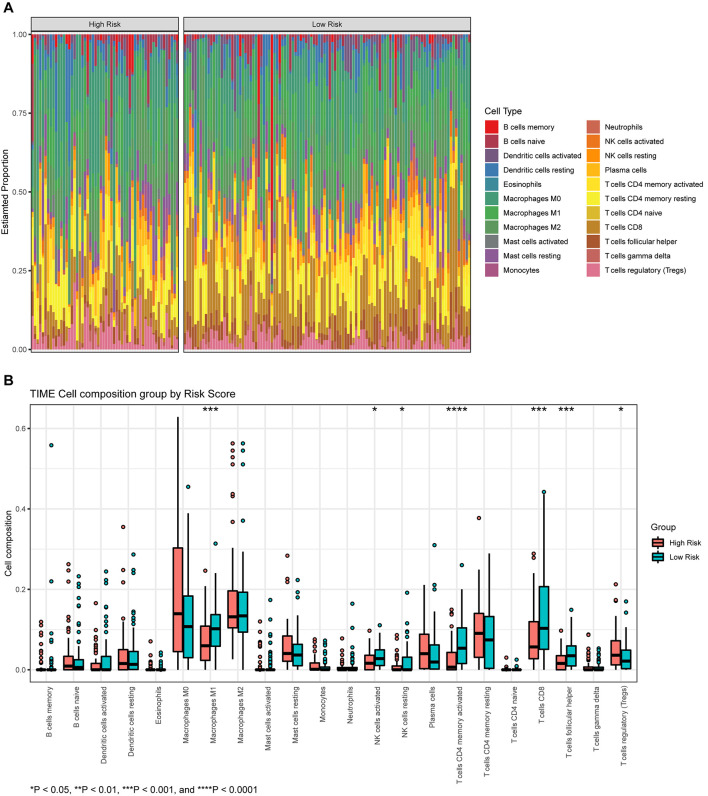
**(A)** The proportion of infiltrating immune cells, as estimated by the CIBERSORT algorithm. **(B)** A box plot of immune cells in the low- and high-risk groups.

The proportions of 22 types of immune cells were determined for different groups of BLCA patients (Figure 8A). We then compared the abundances of tumor-infiltrating immune cells in groups with different levels of risk scores (Figure 8B). The infiltrating levels of resting NK cells, activated natural killer (NK) cells, CD8^+^ T cells, activated memory CD4^+^ T cells, and T follicular helper cells, were significantly higher in the low-risk group. The high-risk group showed predominant infiltration by regulatory T cells (Tregs).

## Discussion

BLCA is associated with morbidity and mortality rates of 3.0 and 2.1% worldwide, respectively (Sung et al., 2021). Because of the diverse range of biological mechanisms that exist between NMIBC and MIBC, it is important that we consider whether to provide different treatment strategies. Previous studies have established multiple prognostic models (Chen et al., 2021; Xu et al., 2021). Furthermore, Yan et al. (2020) built an eight-gene signature to predict OS in patients with BLCA using WGCNA. Qiu et al. (2020) and Shen et al. (2020) also constructed prognostic models based on immune-associated genes; however, none of these established prognostic models were based on invasiveness. Identifying biomarkers relating to the invasiveness of BLCA and using these biomarkers to establish a prognostic model may provide new options for treatment selection and the prediction of prognosis.

In the present study, we aimed to identify signature genes associated with invasiveness and construct a prognostic model based on these signature genes. In total, we identified 1245 DEGs, thus illustrating clear differences in gene expression between NMIBC and MIBC. A specific module, containing 240 genes, was identified by WGCNA, and found to be significantly associated with invasiveness. Several vital pathways were revealed by functional enrichment analysis. Three hub genes (*VSIG2*, *PPFIBP2*, and *DENND2D*) were extracted from this module; two of these genes (*PPFIBP2*, *DENND2D*) played an important role in prognosis as protective factors. We established a prognostic model and presented this model as a nomogram based on the prognosis-associated signatures. Further validation of the nomogram, with calibration curves and time-dependent ROC curves, suggested an acceptable level of accuracy. As well as identifying new biomarkers related to invasiveness and developing a prognostic model, we also used TIME to investigate potential treatment options.

The midnight-blue module was mainly associated with metabolic-related activities, showed by GO functional analysis; this was consistent with the results derived from KEGG pathway enrichment analysis. Analyses showed that several pathways were enriched, including epithelial cell differentiation, and negative regulation of transforming growth factor beta receptor signaling pathway that inhibits the progression of tumor metastasis, as described previously (Tan et al., 2017; Lyu et al., 2019). The current analyses also identified enrichment in positive regulation of GTPase activity, thymic T cell selection, and extracellular exosome, etc.

Three hub genes (*VSIG2*, *PPFIBP2*, and *DENND2D*) were identified as biomarkers that showed upregulation in patients with NMIBC and downregulated in patients with MIBC. *VSIG2* is expressed in the thymus and may be related to antigen presentation (Kariuki et al., 2010). The function of *VSIG2* in cancer has not been described previously, although this gene may serve as a potential biomarker for BCLA. The *PPFIBP2* product is known to be associated with axon guidance and the development of neuronal synapses (Iyama et al., 2017). The protein encoded by *PPFIBP2* is liprin-β2; previous studies have shown that low levels of liprin-β2 is associated with a poor prognosis for urothelial, renal, prostate, lung, head, and neck cancers (Tan et al., 2008; Wu et al., 2018). Tumor cell migration, invasion, and progression, are controlled by the ERK pathway (Ueoka et al., 2000; Reddy et al., 2003). ERK2 is a major facilitator of cell migration and invasion within the tumor microenvironment; liprin-β2 represents a specific target for ERK2. Liprin-β2 can inhibit cell migration and invasion and acts downstream of ERK2. Consequently, liprin-β2 may act by facilitating the transporting of anti-migratory molecules or by halting the recycling of pro-invasive molecules. ERK2 is also known to drive invasiveness by inhibiting liprin-β2 (von Thun et al., 2012). *DENND2D* is considered to act as a tumor suppressor gene and has been implicated in several types of cancer, including hepatocellular, lung, esophageal, and gastric cancer (Ling et al., 2013; Hibino et al., 2014; Kanda et al., 2014, 2015). *DENND2D* is a regulator of Rab GTPases and is highly associated with carcinogenesis and the progression of cancer. In addition, several Rab GTPase family members are known to influence the secretion of exosomes *via* the trans-Golgi network or inducible vesicular transporting (Ponnambalam and Baldwin, 2003; Ostrowski et al., 2010). Adjacent cells can take up exosomes derived from cancer cells, which are able to induce pathways that are involved in the initiation and progression of cancer (Rink et al., 2005; Henderson and Azorsa, 2012). MiR-1246 in tumor exosomes can directly target and downregulate *DENND2D*, as reported previously (Sakha et al., 2016). *DENND2D* is also involved in the miR-522-induced migration and invasion of non-small cell lung cancer cells by targeting *DENND2D* (Zhang et al., 2016). Higher expression levels of *PPFIBP2* and *DENND2D* are known to be associated with lower levels of tumor invasiveness and a better prognosis; our present findings were consistent with these earlier observations.

TIME plays a key role in tumor initiation and progression; furthermore, immunotherapy is often performed as a component of neoadjuvant therapy (Lyu et al., 2020). We assessed tumor immune cell infiltration to explore potential therapies and other prognostic factors, and we then investigated the differences in TIME between different groups. The proportions of resting NK cells, activated NK cells, CD8^+^ T cells, activated memory CD4^+^ T cells, and Tfh cells, were significantly higher in the low-risk group, thus indicating a better prognosis. In contrast, the proportion of Tregs was lower in the low-risk group than in the high-risk group. NK cells are known to kill adjacent cells that express surface markers that are associated with oncogenic transformation (Shimasaki et al., 2020). By secreting cytokines and chemokines, NK cells may induce T cell infiltration and inflammation; they may also prevent metastasis by eliminating circulating tumor cells (Fauriat et al., 2010; Gooden et al., 2011; Lopez-Soto et al., 2017; Malmberg et al., 2017). In pancreatic cancer, memory CD4^+^ T cells are closely related to gemcitabine resistance (Gu et al., 2020). A similar association may exist for BLCA, although further validation is still needed. Patients with a high proportion of CD8^+^ T cells are more likely to show a favorable response to neoadjuvant chemotherapy (Green et al., 2017). In triple-negative breast cancer, tumors with high levels of infiltrating CD8^+^ T cells and memory CD4^+^ T cells might result in a better prognosis (Matsumoto et al., 2016; Oshi et al., 2020); this is consistent with BLCA. A high abundance of CD8^+^ T cells is closely related to high expression levels of multiple immune checkpoint molecules, thus implying that treatment involving immune checkpoint inhibitors may be effective (Oshi et al., 2020). Tfh cells are an independent subset of CD4^+^ T cells derived from naïve T cells that localize to lymphoid follicles and mediate the selection, proliferation, and survival of B cells to generate antibody signals (Eivazi et al., 2016). Tregs constitutively express CTLA-4 and are able to suppress the activation of leukocytes and maintain immune homeostasis (Lu et al., 2017). Patients with high levels of Tregs infiltration may respond effectively to therapy involving ipilimumab and tremelimumab (Simeone et al., 2014; He et al., 2017).

In summary, we successfully identified signature genes associated with invasiveness and used these genes to establish a reliable prognostic model for BLCA. These gene signatures represent potential biomarkers and targets for prognosis and treatment. Risk score acted as an independent prognostic factor and could guide the selection of therapy involving immune checkpoint inhibitors. However, there were some limitations to this study that need to be considered. First, further laboratory experiments are still required to validate the potential mechanisms underlying the action of the signature genes and TIME. Second, our sample size was not sufficient; therefore, we were unable to detect additional risk signatures associated with invasiveness in patients with BLCA.

## Conclusion

In conclusion, we identified three signature genes associated with the invasiveness of BLCA; two of these showed strong associations with prognosis. We also constructed a prognostic risk model that featured the three signature genes and other clinical features; this model showed acceptable levels of performance. Differences in TIME between the patient groups showing different risk scores were also analyzed to guide the selection of therapeutic approaches and to help predict prognosis.

## Data Availability Statement

Publicly available datasets were analyzed in this study. This data can be found here: https://www.ncbi.nlm.nih.gov/geo/ and https://portal.gdc.cancer.gov/.

## Ethics Statement

Ethical review and approval was not required for the study on human participants in accordance with the local legislation and institutional requirements. Written informed consent for participation was not required for this study in accordance with the national legislation and the institutional requirements.

## Author Contributions

YH and AX designed this study. YH obtained and analyzed the data. YH, AX, YW, ZL, BL, NJ, and PX helped to review relevant literature and discuss the results. YH wrote the manuscript. All authors approved the final submitted manuscript.

## Conflict of Interest

The authors declare that the research was conducted in the absence of any commercial or financial relationships that could be construed as a potential conflict of interest.

## Publisher’s Note

All claims expressed in this article are solely those of the authors and do not necessarily represent those of their affiliated organizations, or those of the publisher, the editors and the reviewers. Any product that may be evaluated in this article, or claim that may be made by its manufacturer, is not guaranteed or endorsed by the publisher.

## Footnotes

1
https://www.ncbi.nlm.nih.gov/geo/

2
https://portal.gdc.cancer.gov/

3
http://www.bioconductor.org/

4
https://david.ncifcrf.gov

## Supplementary Material

Supplementary Figure 1 The relationships between modules and the invasiveness of BLCA.

Supplementary Table 1 All genes from the midnight-blue module and their expression levels in each sample.

Supplementary Table 2 The identified hub genes and their MM in each module.

Supplementary Table 3 Clinical information of BLCA samples in TCGA.
