# Corrigendum: Identification of Signature Genes Associated With Invasiveness and the Construction of a Prognostic Model That Predicts the Overall Survival of Bladder Cancer

**DOI:** 10.3389/fgene.2022.880633

**Published:** 2022-05-17

**Authors:** Yang He, Yongxin Wu, Zhe Liu, Boping Li, Ning Jiang, Peng Xu, Abai Xu

**Affiliations:** ^1^ Department of Urology, Zhujiang Hospital, Southern Medical University, Guangzhou, China; ^2^ Department of Urology, The First People’s Hospital of Kashgar Prefecture, Kashgar, China

**Keywords:** bladder cancer (blca), invasiveness, weighted gene co-expression network analysis (WGCNA), nomogram, tumor immune microenvironment

In the original article, there were several errors. The sentence “the risk score had the greatest weighing in this calculation” was repeated two times. In the **Discussion** section, the word “Higher” was incorrectly written as “Lower”. The number “4" was incorrectly superscript applied in the sentence “T cells and memory CD^4+^ T cells might result in a better prognosis.”

A correction has been made to **Results**, “*Construction and Validation of a Nomogram for Predicting Prognosis of Patients With Bladder Cancer*,” *Paragraph 1*:

“Consequently, 1- and 5-year OS can be estimated according to the total number of points; the risk score had the greatest weighing in this calculation.”

A correction has been made to **Discussion**, *Paragraph 4*:

“Higher expression levels of *PPFIBP2* and *DENND2D* are known to be associated with lower levels of tumor invasiveness and a better prognosis; our present findings were consistent with these earlier observations.”

A correction has been made to **Discussion**, *Paragraph 5*:

“In triple-negative breast cancer, tumors with high levels of infiltrating CD8^+^ T cells and memory CD4^+^ T cells might result in a better prognosis;”

The authors apologize for these errors and state that these do not change the scientific conclusions of the article in any way. The original article has been updated.

