# Supplementary figures and images for: Identification of Signature Genes Associated With Invasiveness and the Construction of a Prognostic Model That Predicts the Overall Survival of Bladder Cancer
Source: Front Genet. 2021 Sep 13;12:694777. doi: 10.3389/fgene.2021.694777 (PMC8473900; doi:10.3389/fgene.2021.694777)

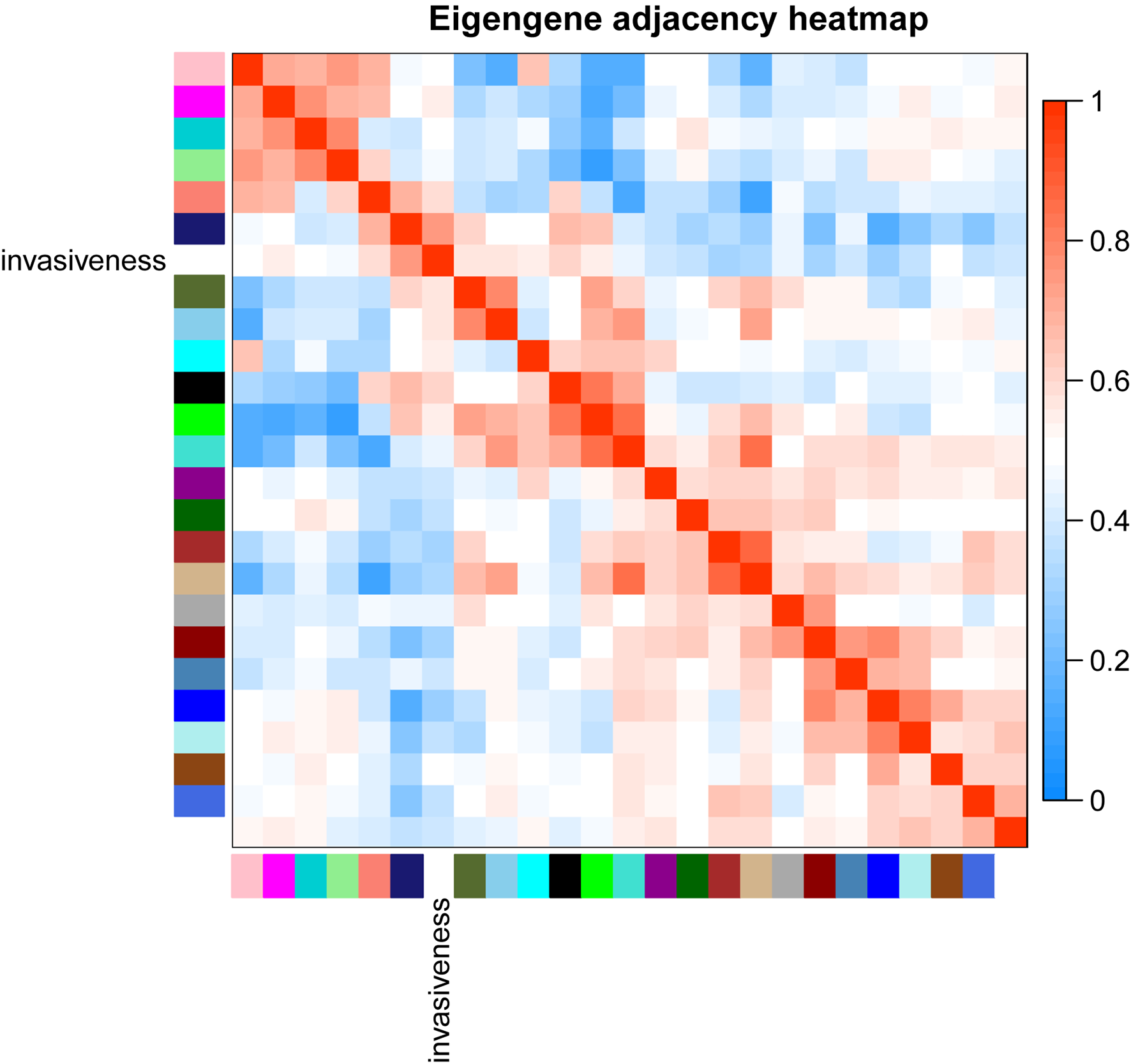

Supplement: Supplementary Figure 1 — The relationships between modules and the invasiveness of BLCA. [file Image_1.TIF]
